# Supplementary material for: Less is more: modelling the impact of species-targeted versus broadcast larviciding in Anopheles funestus-dominated malaria transmission settings
Source: Malar J. 2026 Jul 1;25:338. doi: 10.1186/s12936-026-06018-4 (PMC13591914; doi:10.1186/s12936-026-06018-4)
Supplement: Supplementary file 1 [file 12936_2026_6018_MOESM1_ESM.docx]

**Figure S1.** Impact of combined ITN and larviciding interventions on EIR and the incidence of malaria among children under five. The analysis considers two different larviciding campaign start times: the rainy season (February) and the dry season (August). Various scenarios were modelled with a campaign duration of four months, with fortnightly habitat larviciding and coverage levels. To assess the impact of larviciding, reductions in EIR and malaria incidence were calculated by comparing each intervention scenario to its corresponding ITN-only baseline scenario. A baseline of 40% ITN coverage without larviciding was used for scenarios combining 40% ITN coverage with larviciding, while a baseline of 80% ITN coverage without larviciding was used for scenarios combining 80% ITN coverage with larviciding.

***Assessment of insecticide susceptibility of malaria vectors***

Susceptibility of malaria vectors to insecticide was assessed according to the WHO standard operating procedures [1] between January and August 2024. Mosquitoes for these tests were collected in the study villages. Due to the availability of mosquito larvae, *An. funestus* larvae were collected from Ebuyu and Chirombola, while *An. arabiensis* were collected from Kichangani. Mosquito larvae were then reared in the field (*An. funestus*) or laboratory (*An. arabiensis*) until emergence. Upon emergence, mosquitoes were placed in netting cages and maintained on a sugar solution. Three to five days emerged mosquitoes were used in the susceptibility tests using 0.75% permethrin, 3.75% permethrin, 4% DDT, 0.1% bendiocarb, 0.1%, and 0.25% pirimiphos methyl, a combination of 0.75% permethrin and 4% piperonyl-butoxide (PBO) [2]. Up to 150 mosquitoes were equally placed in six holding tubes for an hour, after which 100 mosquitoes were placed in four test tubes containing insecticide-impregnated papers and 50 were placed in two control tubes containing oil-impregnated papers. After an hour of exposure, knockdown was recorded for each tube, and the mosquitoes were transferred back to the holding tubes, provided with 10% sugar solution, and observed after 24 hours for survival and mortality.

Insecticide susceptibility was assessed by percent mortality: dead mosquitoes/total introduced. Mortality in exposed mosquitoes was corrected using Abbott’s formula when control mortality was 5-20%. Susceptibility was categorized: ≥98% mortality = susceptible, 90−97.9% = possibly resistant, <90% = resistant [1]. Experiments with control mortality >20% were discarded.

**Figure S2.** Study villages in Ulanga district (south-eastern Tanzania) where mosquito larvae were collected insecticide susceptibility tests.

***Insecticide susceptibility of malaria vectors***

Both *An. funestus* and *An. arabiensis* exhibited resistance to permethrin, with mortality rates below 20%. However, *An. funestus* displayed a higher level of resistance, remaining resistant even at a fivefold higher dose of permethrin, while *An. arabiensis* became susceptible (with 100% mortality) to the insecticide at this increased concentration. *An. funestus* maintained a high level of resistance to DDT, with a mortality rate of only 8%. Conversely, *An. arabiensis* remained fully susceptible to DDT, exhibiting a 100% mortality rate. Tests using carbamates indicated a potential for resistance in both species to bendiocarb, with mortalities of 97.9% for *An. funestus* and 96.9% for *An. arabiensis*. Finally, both *An. funestus* and *An. arabiensis* were fully susceptible to the organophosphate pirimiphos methyl. Mortality rates for this insecticide exceeded 98% in both mosquito species (Fig. S2).

**Figure S3.** Percentage mortality of *An. funestus* and *An. arabiensis* exposed to different classes of insecticides. Mortality below the red dotted line (90%) signifies confirmed resistance, mortality between the red dotted line (90%) and grey dotted line (98%) signifies the possibility of resistance, and mortality above the grey dotted line (98%) signifies susceptibility of tested mosquitoes.
